# Personal

**Published:** 1904-05

**Authors:** 


### PERSONAL.

Dr. GEORGE E. BREWER, formerly of Buffalo, and lately professor of clinical surgery at the College of Physicians and Surgeons, Columbia University, has been assigned a seat in the medical faculty of that university. Dr. Brewer is a graduate of the University of Buffalo, class of 1884.

---

Dr. WILLIAM A. EVANS, of Chicago, is among those considered for the presidency of the University of Illinois, vice Dr. A. S.

Draper, appointed commissioner of education of the state of New York. Dr. Evans is a well-known physician of Chicago, 45 years old, and has been practising his profession since he was graduated, in 1885, from Tulane University, Louisiana. He went to Chicago twelve years ago, and in a comparatively short time gained an international reputation through his original researches into many fields of scientific and economic effort. He is an ex-president of the Chicago Medical Society and a leading member of the principal medical bodies of the city. Dr. Evans will be remembered pleasantly by the members of the American Association of Obstetricians and Gynecologists who attended the meeting at Chicago, last September, both for the spirited address of welcome which he delivered upon the opening of the meeting and also for the charming speech made at the banquet.

---

Dr. Roswell Park, of Buffalo, who has been spending a few weeks in Europe, will return and resume his surgical practice about May 10, 1904.

---

Dr. H. B. Brownell, of Buffalo, was one of a group of physicians who sailed for Europe in the latter days of April.

---

Dr. Charles W. Banta, of Buffalo, sailed for Europe in company with Dr. Buswell and others April 23, 1904, to remain abroad several months.

---

Dr. F. Whitehill Hinkel, of Buffalo, will remove his office about June 1, 1904, from 412 Franklin street to 581 Delaware avenue. Hours: 9 to 1.

---

Dr. Henry C. Buswell, of Buffalo, sailed for Europe on the Pretoria, April 23, 1904, for an extended tour in Great Britain and on the continent. He will be absent several months.

---

Dr. Henry Y. Grant, of Buffalo, who with Mrs. Grant has been spending the winter in Europe, returned about April 22, and has resumed his ophthalmological practice at 401 Delaware avenue.

---

Dr. Gustav A. Pohl, of Buffalo, removed his office and residence, May 1, 1904, to 621 Oak street near Carlton. Hours: 8.30 to 9.30 a. m., 9 to 2 and 7 to 8 p. m. No Sunday hours. Bell telephone.

Dr. EDWARD J. MEYER, of Buffalo, has gone to Europe to spend a number of months.

---

Dr. C. H. W. AUEL, of Buffalo, has been reëlected president of the medical staff of the German Hospital to serve for the current year.

---

Dr. JOHN O. ROE, of Rochester, has removed his office from 28 Clinton avenue, north, to 44 Clinton avenue, south.

---

Dr. and Mrs. LAWRENCE G. HANLEY, of Buffalo, entertained at dinner at their home on Porter avenue, Saturday evening, April 16, a number of physicians who were on the eve of departure for Europe. The company consisted of Drs. H. C. Buswell, Edward J. Meyer and Charles W. Banta, voyageurs. Other guests were Drs. Rollin L. Banta, Edward T. Smith and John M. Mesmer, Buffalo, and B. E. Smith, Angola. Dr. and Mrs. Hanley expect to join the European party at Berlin in August.

---

Dr. WILLIAM T. BULL, of New York, has resigned the professorial chair of surgery at the College of Physicians and Surgeons, Columbia University, to take effect June 30, 1904. Dr. Bull's action is greatly regretted by his colleagues and others. This step has become necessary on account of the increasing demands of his private practice.

---

Dr. L. S. MCMURTRY, of Louisville, read a paper before the Saint Louis Medical Society by invitation, April 16, 1904.

---

Dr. P. MAXWELL FOSHAY, of Cleveland, has removed to Chicago to take the medical directorship of the western branch of the New York Mutual Life Insurance Company. Dr. Foshay was editor of the *Cleveland Medical Journal,* which he conducted with signal ability. He was one of the committee on reorganisation of the American Medical Association, and achieved distinction in that service. He is one of the earlier licentiates of the New York State Medical Examining and Licensing Board, his license being No. 9. While Dr. Foshay will be missed in Cleveland, where he has been distinguished for activity in all that goes for medical progress, he will receive a cordial welcome in Chicago, where a larger field awaits his energetic labors.

